# Magnolol Ameliorates Ligature-Induced Periodontitis in Rats and Osteoclastogenesis: *In Vivo* and *In Vitro* Study

**DOI:** 10.1155/2013/634095

**Published:** 2013-03-21

**Authors:** Sheng-Hua Lu, Ren-Yeong Huang, Tz-Chong Chou

**Affiliations:** ^1^Graduate Institute of Life Sciences, National Defense Medical Center, Taipei, Taiwan; ^2^Department of Periodontology, School of Dentistry, Tri-Service General Hospital and National Defense Medical Center, Taipei, Taiwan; ^3^Department of Biomedical Engineering, National Defense Medical Center, No. 161, Min-Chuan East Road, Section 6, Taipei, Taiwan

## Abstract

Periodontal disease characterized by alveolar bone resorption and bacterial pathogen-evoked inflammatory response has been believed to have an important impact on human oral health. The aim of this study was to evaluate whether magnolol, a main constituent of *Magnolia officinalis*, could inhibit the pathological features in ligature-induced periodontitis in rats and osteoclastogenesis. The sterile, 3–0 (diameter; 0.2 mm) black braided silk thread, was placed around the cervix of the upper second molars bilaterally and knotted medially to induce periodontitis. The morphological changes around the ligated molars and alveolar bone were examined by micro-CT. The distances between the amelocemental junction and the alveolar crest of the upper second molars bilaterally were measured to evaluate the alveolar bone loss. Administration of magnolol (100 mg/kg, p.o.) significantly inhibited alveolar bone resorption, the number of osteoclasts on bony surface, and protein expression of receptor activator of nuclear factor-**κ**B ligand (RANKL), a key mediator promoting osteoclast differentiation, in ligated rats. Moreover, the ligature-induced neutrophil infiltration, expression of inducible nitric oxide synthase, cyclooxygenase-2, matrix metalloproteinase (MMP)-1 and MMP-9, superoxide formation, and nuclear factor-**κ**B activation in inflamed gingival tissues were all attenuated by magnolol. In the *in vitro* study, magnolol also inhibited the growth of *Porphyromonas gingivalis and Aggregatibacter actinomycetemcomitans* that are key pathogens initiating periodontal disease. Furthermore, magnolol dose dependently reduced RANKL-induced osteoclast differentiation from RAW264.7 macrophages, tartrate-resistant acid phosphatase (TRAP) activity of differentiated cells accompanied by a significant attenuation of resorption pit area caused by osteoclasts. Collectively, we demonstrated for the first time that magnolol significantly ameliorates the alveolar bone loss in ligature-induced experimental periodontitis by suppressing periodontopathic microorganism accumulation, NF-**κ**B-mediated inflammatory mediator synthesis, RANKL formation, and osteoclastogenesis. These activities support that magnolol is a potential agent to treat periodontal disease.

## 1. Introduction

Periodontitis, a chronic infectious inflammatory disease, is highly prevalent among world populations [1]. Recently, it has been generally accepted that periodontal disease is not only an important impact factor to human oral health but also a critical risk factor promoting several systemic diseases [2]. A number of studies have indicated that the main pathogen of periodontal disease is the bacterial plaque accumulation predominantly including *Porphyromonas gingivalis *and *Aggregatibacter actinomycetemcomitans *[3–5]. The bacterial pathogen is known to play a critical role in the initiation of inflammatory processes in periodontal tissues and host immune response by releasing several inflammatory mediators such as proinflammatory cytokines, prostaglandin E_2_ (PGE_2_), and reactive oxygen species (ROS), as well as upregulating periodontitis-related gene expression including inducible nitric oxide synthase (iNOS), cyclooxygenase-2 (COX-2), and matrix metalloproteinases (MMPs) [6–9]. These harmful factors further result in gingivitis, periodontal pocket formation, alveolar bone resorption, and the tooth supporting structure damage and finally lead to tooth loss. Additionally, the increased host inflammatory process in response to bacteria is often accompanied by an uncoupling of bone formation following bone resorption [10]. It has been demonstrated that the alveolar bone loss, a clinical characteristic of periodontitis, is dependent on the balance between bone resorption via osteoclasts and bone formation via osteoblasts. Importantly, the bone resorption is stimulated by the receptor activator of nuclear factor-*κ*B ligand (RANKL), but negatively regulated by its decoy receptor, osteoprotegerin (OPG) [11], suggesting that attenuation of RANKL/OPG ratio may prevent osteoclastogenesis and bone resorption in periodontitis. In addition, suppressing the bacteria-mediated inflammation is a promising target to ameliorate periodontal disease.

Currently, the fundamental treatment of periodontitis is aimed to reduce the pathogenic microbiota by instrumental debridement [12]. However, mechanical instrumentation fails to completely eliminate the pathogenic bacteria that have invaded into soft tissue and anatomically inaccessible areas such as the furcation area and root depression. Owing to these unavoidable limitations, development of potential therapeutic drugs with an ability to regulate the host immune and bacteria-mediated inflammatory interactions is a valuable approach for prevention and treatment of periodontal diseases [13]. Recently, many natural products have been reported to improve the pathological symptoms of periodontitis [14, 15], suggesting that medicinal herbs may be potential drugs for periodontal therapy. Magnolol extracted from *Magnolia officinalis* is widely used in oriental medicine [16]. There is growing evidence indicating that magnolol exhibits a variety of beneficial pharmacological activities including anticancer [17], antiplatelet [18], and antioxidative effects [19]. Moreover, magnolol has been reported to have a potent anti-inflammatory activity via inhibition of proinflammatory cytokine, ROS formation, iNOS, COX-2 expression, and nuclear factor-kappaB (NF-kappaB) activation, a key transcription factor regulating inflammation, in LPS-induced inflammatory diseases [20, 21]. Furthermore, magnolol exerts a marked antimicrobial activity against periodontopathic bacteria [22] and activates osteoblast function [23]. Based on these properties, magnolol may be a potential candidate for treating periodontitis. However, there is little information about the effect of magnolol on the pathogenesis of periodontitis. Therefore, the present study was conducted to determine whether magnolol can inhibit the inflammatory responses and prevent the alveolar bone loss in ligature-induced experimental periodontitis in rats.

## 2. Materials and Methods

### 2.1. Animals and Induction of Experimental Periodontitis

Males Sprague-Dawley rats (250–350 g) purchased from Biolasco, Inc., Taiwan, were housed in Laboratory Animal Center of National Defense Medical Center under a 12-h light/dark cycle with temperature at 21 ± 1°C and humidity at 40–60%. The rats had free access to tap water and standard food. All experiments were conducted in accordance with guidelines for the welfare of experimental animals and were approved by the Institutional Animal Care and Use Committee (IACUC), National Defense Medical Center, Taipei, Taiwan. After rats were anesthetized with sodium pentobarbital (35 mg/kg, i.p.), the sterile, 3–0 (diameter; 0.2 mm) black braided silk thread (surgical silk sutures; UNIK, Taipei, Taiwan), was placed around the cervix of the upper second molars bilaterally and knotted medially to induce periodontitis as previously described [24]. The ligatures were gently displaced apically into the gingival sulci under a stereomicroscope to ensure a subgingival position, and replaced when dislodged or lost. For *in vitro* tests, magnolol (Sigma, St. Louis, MO, USA) was dissolved in dimethylsulfoxide (DMSO) followed by dilution, and the final concentration of DMSO was fixed at 0.1%.

### 2.2. Experimental Groups

Animals were randomly allocated into three groups with five rats per group. The control group did not receive the induction of periodontitis and was given vehicle (normal saline) alone. The rats of ligation group were subjected to ligature placement and administrated with vehicle. Rats of ligation + magnolol group received the same ligature placement and treated daily with magnolol (100 mg/kg) by oral gavage for 9 days starting 1 day before ligature. Rats were sacrificed by carbon dioxide inhalation at 8 days after ligature, and specimens and the gingival tissues encircling the ligated molars were taken for further analysis.

### 2.3. Micro-Computerized Tomography (Micro-CT) Imaging

The morphology around the ligated molars and alveolar bone of various groups (5 rats/group) was examined by micro-CT in all dimensions as previously described [24]. The distances between the amelocemental junction (ACJ) and the alveolar crest (AC) of both buccal and palatal sides of the upper second molars bilaterally were measured by using VIVID software (Gamma Medica-Ideas, Northridge, CA, USA). The average of the ACJ-AC distances was used to evaluate the alveolar bone loss.

### 2.4. Histological and Histometric Analyses

The specimens (including gingivae, teeth, and bones) around the molars were dissected, fixed in 10% buffered neutral formalin for 48 h, and decalcified in 10% ethylenediaminetetraacetic acid (EDTA) (Sigma, St Louis, MO, USA) for 4 weeks. Each sample was embedded in paraffin wax and sliced into 4 *μ*m thick sections in mesiodistal directions. Sections were mounted on glass slides and stained with hematoxylin and eosin (H&E), and the number of infiltrated inflammatory polymorphonuclear neutrophil (PMN) cells in gingivae was calculated by ImageJ image analysis and process software (National institutes of Health, USA). The osteoclasts were identified by tartrate-resistant acid phosphatase (TRAP) staining near to the bony resorptive lacunae and multiple nuclei (≧three).

### 2.5. Preparation of Periodontal Pathogens and Antibacterial Assay

The* Porphyromonas gingivalis* ATCC 33277 and *Aggregatibacter actinomycetemcomitans *ATCC 29522 purchased from Bioresource Collection and Research Center (Hsinchu, Taiwan) were grown on brain heart infusion (BHI) agar (BD, Franklin Lakes, NJ, USA) under an anaerobic chamber at 37°C for 1 day [18]. Then, the bacteria were harvested, suspended in brain heart infusion (BHI) broth, and grown to a density of 1 × 10^5^ colony forming units per milliliter (CFU/mL). Vehicle (0.1% DMSO) or various concentrations of magnolol were added into the bacterial solution for 24 h. The optical densities (O.D) of the bacterialsolution were measured at 600 nm and further determined the half-maximal inhibitory concentration (IC_50_) to evaluate the antibacterial activity of magnolol.

### 2.6. Measurement of Myeloperoxidase (MPO) Activity

Myeloperoxidase activity, an index of neutrophil accumulation, in gingivomucosal tissues was determined. Gingivomucosal tissues surrounding the bilateral upper second molars were dissected and were homogenized in a solution containing 50 mM potassium phosphate, (pH 6) containing 5 mM hexadecyltrimethylammonium bromide (Sigma-Aldrich, St. Louis, MO, USA), and subjected to three cycles of freezing and thawing followed by centrifugation at 14,000 g for 10 min at 4°C to obtain the supernatant. MPO activity was determined by combining 20 *μ*L tissue supernatant with 40 *μ*L assay buffer containing 0.167 mg/mL O-dianisidine dihydrochloride (Sigma-Aldrich, St. Louis, MO, USA) and 0.0005% hydrogen peroxide. The MPO activity was calculated as the change in absorbance at 460 nm over 1 min and expressed as U/g weight.

### 2.7. Determination of Superoxide Anion (O_2_^−^) Production

The gingivomucosal tissues were collected, sliced into pieces, and incubated with warmed (37°C), oxygenated (95% O_2_/5% CO_2_) Krebs-Hepes buffer for 5–10 min. Then, the samples were transferred to 96-well microplates containing 200 *μ*L Krebs-Hepes buffer with 50 *μ*L lucigenin (1.25 mM) and counted by a microplate luminometer (Microlumat Plus LB96V, Berthold, Germany) at room temperature. The O_2_^−^ production was expressed as count per second (CPS) per milligram of dry tissue weight (CPS/mg dry weight).

### 2.8. Western Blotting

To enhance the extractive efficiency, the gingival tissues were excised and homogenized by precellys 24 homogenizer (Bertin Technologies). Then, the protein extracts were separated on 12% SDS-polyacrylamide gels (SDS-PAGE) and transferred to nitrocellulose membranes. The membranes were subsequently blocked by tris-buffered saline (TBS) containing 5% powered nonfat milk for 1 h and then incubated with appropriately diluted primary target antibodies for overnight at 4°C. The membranes were washed and incubated with horseradish peroxidase-conjugated secondary antibody for 1 h, and the immunoreactivity was visualized using enhanced HRP Substrate Luminol Reagent (Millipore, Billerica, MA, USA). The relative intensity of blotted protein was measured densitometrically with ImageJ image software.

### 2.9. Cell Viability Assay

The mouse RAW264.7 macrophages obtained from Bioresource Collection and Research Center (Hsinchu, Taiwan) were grown in DMEM (Sigma, St Louis, MO, USA) supplemented with 10% heat-inactivated FBS (Gibco, NY, USA). After incubation of magnolol (20 *μ*M) with cells for 24 h, the cell viability was determined by assay of the reduction of 3-[4,5-dimethylthiazol-2-yl]-2,5-diphenyl tetrazolium bromide (MTT) to formazan and measured the absorbance at 570 nm.

### 2.10. Osteoclast Differentiation

Osteoclast differentiation and TRAP activity were performed as previously described [25]. Briefly, RAW 264.7 cells were seeded at 5 × 10^5^ cells/well in a 24-well plate. After incubation for 24 h, the media were replaced and the cells were cultured for an additional 4 days in *α*-minimal essential medium (*α*-MEM) (Sigma, St Louis, MO, USA) supplemented with 10% FBS and RANKL (50 ng/mL) (Peprotech, Rocky Hill, NJ, USA) in the presence of magnolol (2.5–20 *μ*M). Then, the cells were fixed with 10% formalin for 10 min and ethanol/acetone (1 : 1) for 1 min, followed by staining with TRAP (Sigma, St Louis, MO, USA). The images of TRAP-positive multinucleated cells (MNCs ≧three nuclei) were captured and counted under a light microscope (Leica, Vertrieb Deutschland, Germany) to evaluate the osteoclast differentiation. For the determination of TRAP activity, the cells were lysed and incubated with 50 mM citrate buffer (pH 4.6) containing 10 mM tartrate and 5 mM p-nitrophenylphosphate (pNPP) for 1 h. The reaction was stopped by addition of 0.1 N NaOH and analyzed spectrophotometrically at 410 nm.

### 2.11. Pit Formation Assay

For pit formation assay, RAW 264.7 cells (1 × 10^5^ cells/well) were seeded on Corning Osteo Assay Surface well (Corning, NY, USA) and cultured in *α*-MEM medium according to the manufacturer's instructions. The cells were pretreated with various concentrations of magnolol (5–20 *μ*M) or vehicle (0.1% DMSO) 2 h before the addition of RANKL (50 ng/mL) and incubation for 4 days. Then, the cells were removed with 1 N NaOH for 20 min, and the wells were washed twice with PBS. The resorption areas were visualized by microscopy (Leica, Vertrieb Deutschland, Germany) and analyzed by Metamorph imaging analysis software (Downingtown, PA, USA).

### 2.12. Statistical Analysis

Data are expressed as means and their standard deviation. Statistical analysis between groups was made by using one-way ANOVA followed by Bonferroni test. Differences were considered significant at *P* < 0.05.

## 3. Results

### 3.1. Magnolol Decreases Alveolar Bone Loss in Experimental Periodontitis

**Figure 1:**
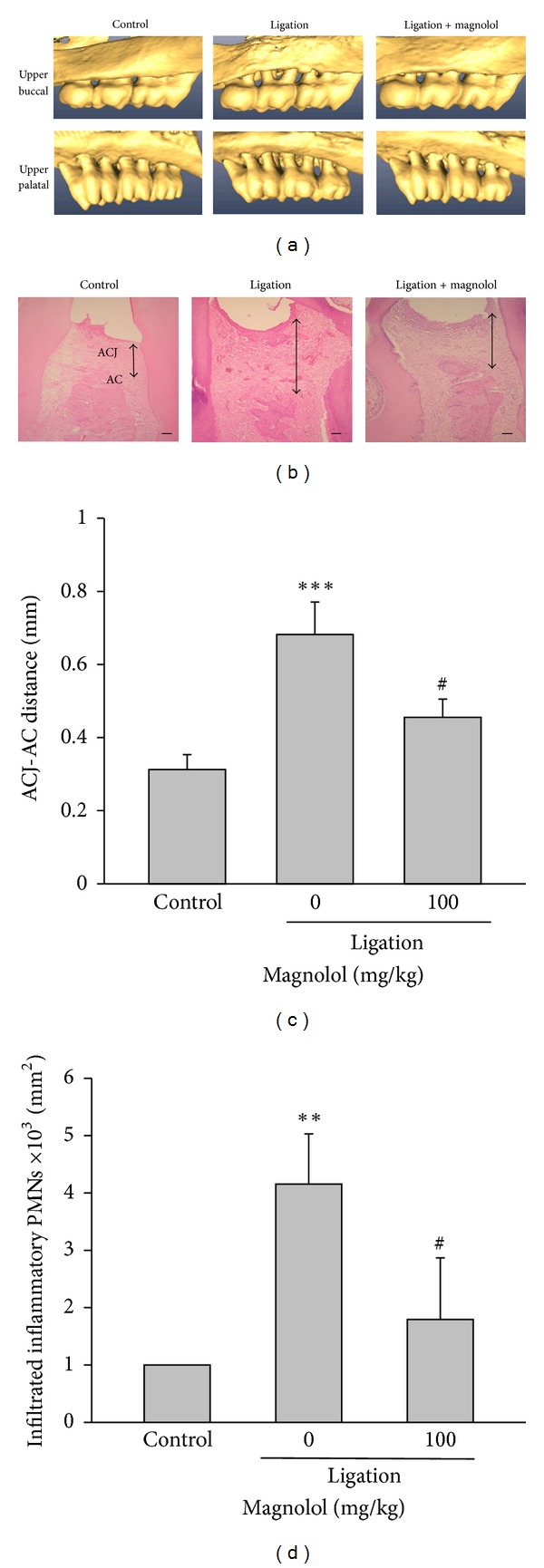
Effect of magnolol on alveolar bone loss and tissue damage. (a) Reconstructed three-dimensional micro-CT images (buccal and palatal view) of the maxilla second molars buccal (upper panel) and palatal (lower panel) alveolar bone level in nonligation (control), ligation, and ligation + magnolol (100 mg/kg) groups at the experimental day 8. (b) Histological observation (H&E stain) of maxillary intermolar tissue in various groups (scale bar = 100 *μ*m). (c) The degree of alveolar bone loss was evaluated by measuring the ACJ-AC distance at the buccal furcation site of the maxilla second molar from the reconstructed micro-CT images. (d) Quantitative analysis of the total number of inflammatory infiltrated cells in maxillary intermolar gingivomucosal tissue was performed by accounting the number of polymorphonuclear cells (PMNs). Data are expressed as the mean ± SD (*n* = 5). ***P < 0.001 versus control; ^#^ *P* < 0.05, ^##^ *P* < 0.01 versus ligation group.

The alveolar bone loss of various groups was assessed by micro-CT assay. Our results showed that there was a severe bone resorption evidenced by a marked increase of ACJ-AC distances both at buccal and palatal aspects in the ligation group when compared to the nonligation (control) group (Figures 1(a) and 1(c)). Consistently, the histopathological analysis of the region between the first and the second molars indicated a remarkable loss of connective tissue of periodontal architecture in ligation group compared to the control group (Figure 1(b)). In addition, a greatly increased inflammatory cell infiltration was observed in rats subjected to ligature-induced periodontitis when compared to the control group (Figure 1(d)). Administration of magnolol (100 mg/kg, p.o) significantly diminished ligature-induced bone loss, histological changes, and inflammatory cell infiltration in the experimental periodontitis. Furthermore, the signs of inflammation in gingival tissue around the ligated molars were reduced in rats of ligation + magnolol group. These results clearly indicate that magnolol exerts a protective effect against bone resorption and inflammation in ligature-induced periodontitis. In addition, there was no difference in alveolar bone structure between nonligature group and nonligature plus magnolol (100 mg/kg) treatment group (data not shown), indicating that magnolol alone did not affect the alveolar bone architecture.

### 3.2. Magnolol Suppresses the Number of TRAP-Positive Cells and RANKL Expression

**Figure 2:**
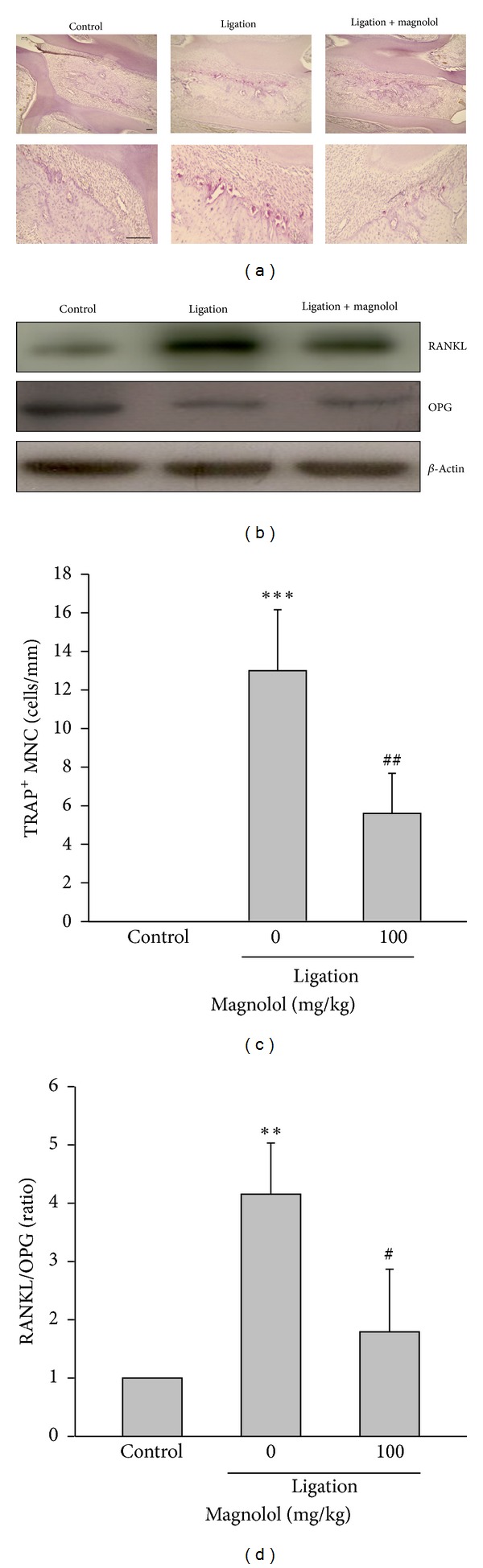
Effect of magnolol on the number of TRAP-positive multinucleated cells (MNCs) and the expression of RANKL and OPG in gingival tissues. (a) The micrographs (upper panel: 100x magnification; lower panel: 200x magnification) represent the histological sections around intermolar region of rats 8 days after ligature placement, and the quantitative analysis of the number of TRAP^+^ MNCs with red staining along the bony resorptive lacunae (cells/mm) was performed (scale bar = 50 *μ*m) (c). (b) The protein expression of RANKL and OPG was determined by Western blot in dissected tissues, (d) and the relative protein RANKL/OPG ratio was measured. Data are expressed as mean ± SD (*n* = 5). **P < 0.01, ***P < 0.001 versus control; ^#^ *P* < 0.05, ^##^ *P* < 0.01 versus ligation group.

At 8 days after ligation, the histological assay showed that the number of osteoclasts stained with TRAP along the bony surface was markedly increased in ligated rats, which was strongly inhibited by magnolol treatment (Figures 2(a) and 2(c)). It is known that RANKL is a key mediator promoting osteoclast differentiation. Adversely, OPG, a natural inhibitor of RANKL, possesses a negative effect on the differentiation of osteoclasts. Our data showed that the RANKL expression in rat gingival tissues was markedly higher in ligation group than that of control group. As expected, administration of magnolol significantly inhibited ligature-induced RANKL expression (Figure 2(b)). Conversely, compared to control group the protein level of OPG in the gingival tissues was reduced in the ligation group, which was not affected by magnolol (Figure 2(b)). Consequently, the RANKL/OPG protein ratio in gingival tissues was remarkably increased in ligation group, whereas the elevated ratio was significantly attenuated by magnolol (Figure 2(b)). These findings suggest that magnolol has an ability to inhibit osteoclast formation and subsequent bone loss by suppressing RANKL expression.

### 3.3. Antibacterial Activity of Magnolol on Periodontopathic Bacteria

**Figure 3:**
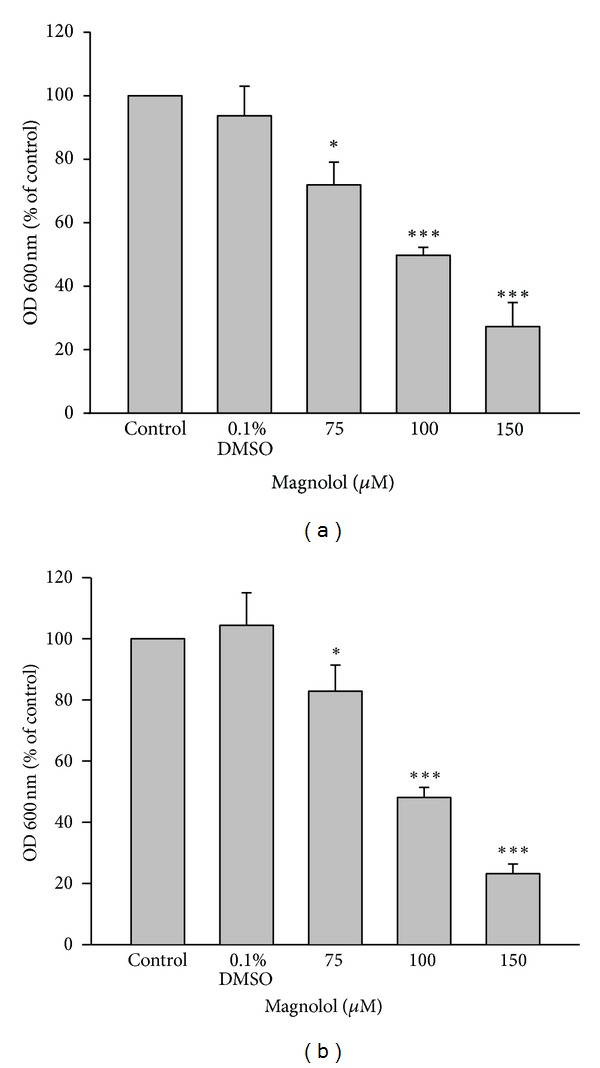
Effect of magnolol on *Porphyromonas gingivalis* and *Aggregatibacter actinomycetemcomitans* growth *in vitro*. Calibrated suspensions of the periodontal pathogens, *Porphyromonas gingivalis* (a) and *Aggregatibacter actinomycetemcomitans* (b), were incubated with medium (control), vehicle (0.1% DMSO), and indicated concentration of magnolol (75–150 *μ*M) for 24 h. The growth of the bacteria was measured with a spectrophotometer at 600 nm wavelength. Data are expressed as the mean ± SD (*n* = 5). *P < 0.05, **P < 0.01, and ***P < 0.001 versus control.

Addition of magnolol (75–150 *μ*M) resulted in a marked inhibition on *Porphyromonas gingivalis* (Figure 3(a))* and Aggregatibacter actinomycetemcomitans *(Figure 3(b)) growth in a dose-dependent manner. The values of IC_50_ of magnolol on the two bacteria were about 100 *μ*M.

### 3.4. Magnolol Inhibits Myeloperoxidase (MPO) Activity, O_2_^−^ Formation, and Expression of COX-2 and iNOS

**Figure 4:**
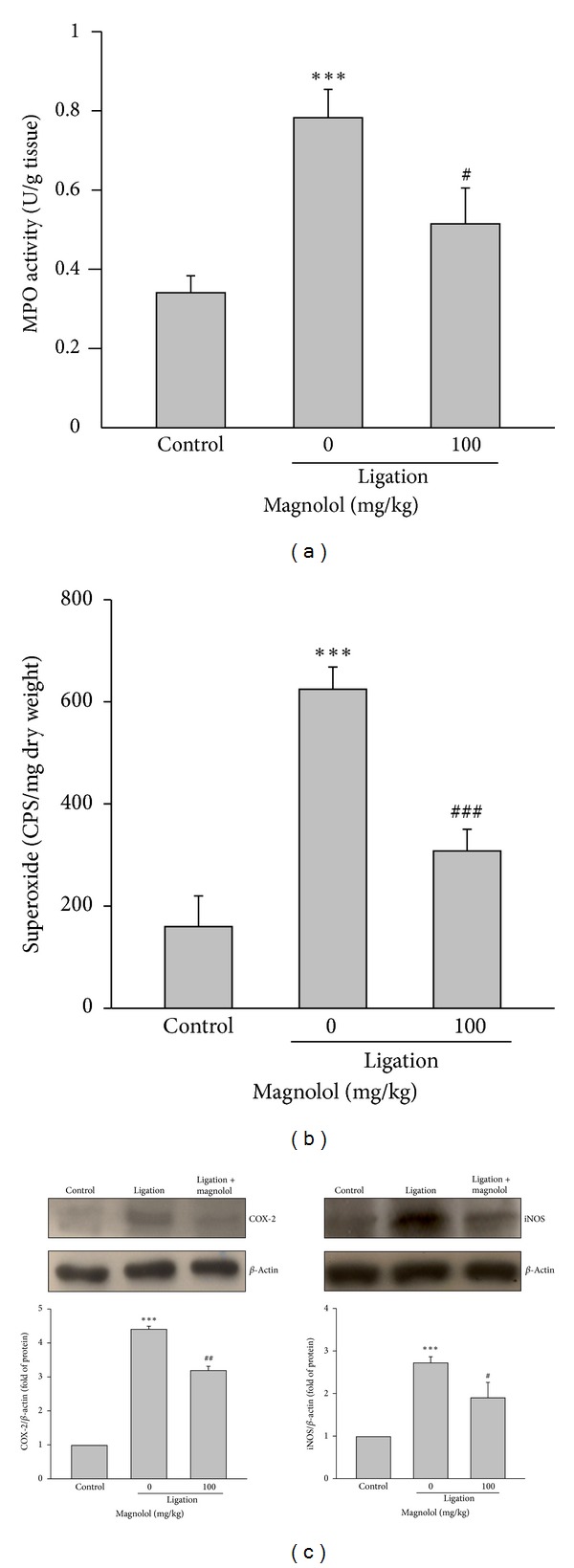
Effect of magnolol on MPO activity, superoxide anion production, and expression of COX-2 and iNOS in inflamed periodontal tissues. (a) The MPO activity and (b) superoxide anion production were determined in the gingivomucosal tissues of each experimental group. (c) The Western blot analysis was used to detect COX-2 and iNOS protein level in dissected tissues encircled upper second molar. Data are expressed as the mean ± SD (*n* = 5). ***P < 0.001 versus control; ^#^ *P* < 0.05, ^##^ *P* < 0.01, and ^###^ *P* < 0.001 versus ligation group.

The MPO activity and O_2_^−^ production in the gingivomucosal tissues increased greatly in ligation group compared to control group, and the increase was significantly reduced by magnolol treatment (Figures 4(a) and 4(b)). Similarly, the ligature-induced protein expression of COX-2 and iNOS in gingival tissues was markedly inhibited by magnolol (Figure 4(c)).

### 3.5. Magnolol Attenuates the MMP-9/TIMP-1 Ratio and NF-*κ*B Activation

**Figure 5:**
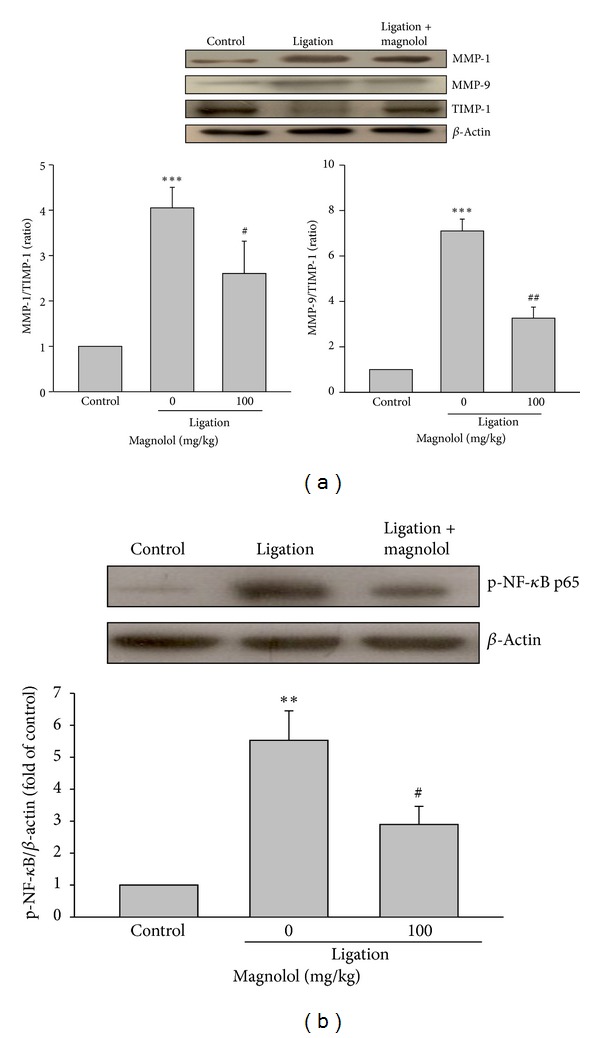
Effect of magnolol on MMP-1, -9, and TIMP-1 expression and NF-*κ*B activation. (a) The protein expression of MMP-1, -9, and TIMP-1 was determined by Western blot in dissected tissues encircled upper second molar, and the relative MMPs/TIMP ratio was measured. (b) The Western blot analysis was conducted to detect phospho-NF-*κ*B p65 protein level in dissected tissues. Data are expressed as the mean ± SD (*n* = 4). **P < 0.01, ***P < 0.001 versus control; ^#^ *P* < 0.05, ^##^ *P* < 0.01 versus ligation group.

The enhanced protein expression of MMP-1 and MMP-9 in inflamed gingival tissues of ligated rats was reduced by the administration of magnolol (100 mg/kg). On the contrary, the level of tissue inhibitor of metalloproteinases-1 (TIMP-1) was decreased in ligation group, which was strongly increased in rats of ligation + magnolol group. Consequently, treatment with magnolol significantly attenuated the ratios of MMP-1/TIMP-1 and MMP-9/TIMP-1 in the inflamed gingival tissues (Figure 5(a)). It is known that NF-*κ*B activation is essential for several inflammation-related gene expression such as iNOS and COX-2 and osteoclast formation and survival [26, 27]. Our results showed that magnolol significantly inhibited ligature-induced NF-*κ*B activation in gingival tissue evidenced by an attenuation of phosphorylation of NF-*κ*B p65 as compared to ligation group (Figure 5(b)). These findings suggest that the anti-inflammatory activity of magnolol in the experimental periodontitis may be, at least in part, due to inhibition of NF-*κ*B activation.

### 3.6. Magnolol Inhibits RANKL-Induced Osteoclast Formation in RAW264.7 Cells

**Figure 6:**
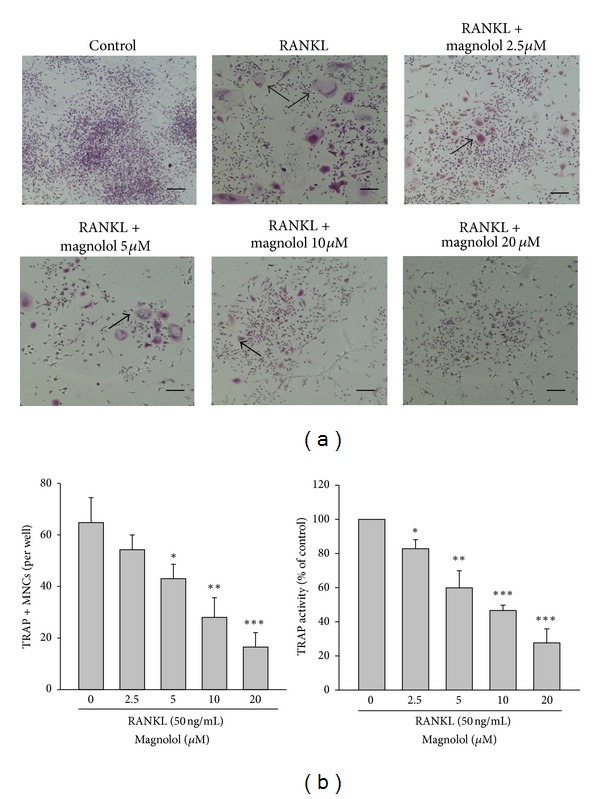
Effect of magnolol on RANKL-induced osteoclast differentiation. RAW 264.7 cells were cultured with the indicated dose of magnolol (2.5–20 *μ*M) in the presence of RANKL (50 ng/mL) for 4 days. The photographs of TRAP-stained osteoclasts (a), the number of TRAP^+^ MNCs, and the TRAP activity (b) were examined (scale bar = 100 *μ*m). Data are expressed as the mean ± SD (*n* = 5). *P < 0.05, **P < 0.01, and ***P < 0.001 versus RANKL-treated group alone.

To gain further mechanistic insights into the actions of magnolol on osteoclast differentiation and function, the effect of magnolol on osteoclast differentiation in the presence of RANKL was investigated *in vitro*. We found that magnolol dose dependently impaired RANKL-induced osteoclastogenesis from RAW264.7 cells reflected by a significant reduction of the number of TRAP-positive multinucleated osteoclasts (TRAP^+^) (Figures 6(a) and 6(b)). Similarly, stimulating with RANKL for 4 days, the enhanced TRAP activity of differentiated RAW 264.7 cells was markedly inhibited by magnolol in a dose-dependent manner (Figure 6(b)). In addition, the effects of magnolol on osteoclast differentiation and function were not due to its cytotoxicity, because no evident cytotoxicity was found under the dose of magnolol used (data not shown).

### 3.7. Magnolol Inhibits RANKL-Induced Bone Resorption in RAW264.7 Cells

**Figure 7:**
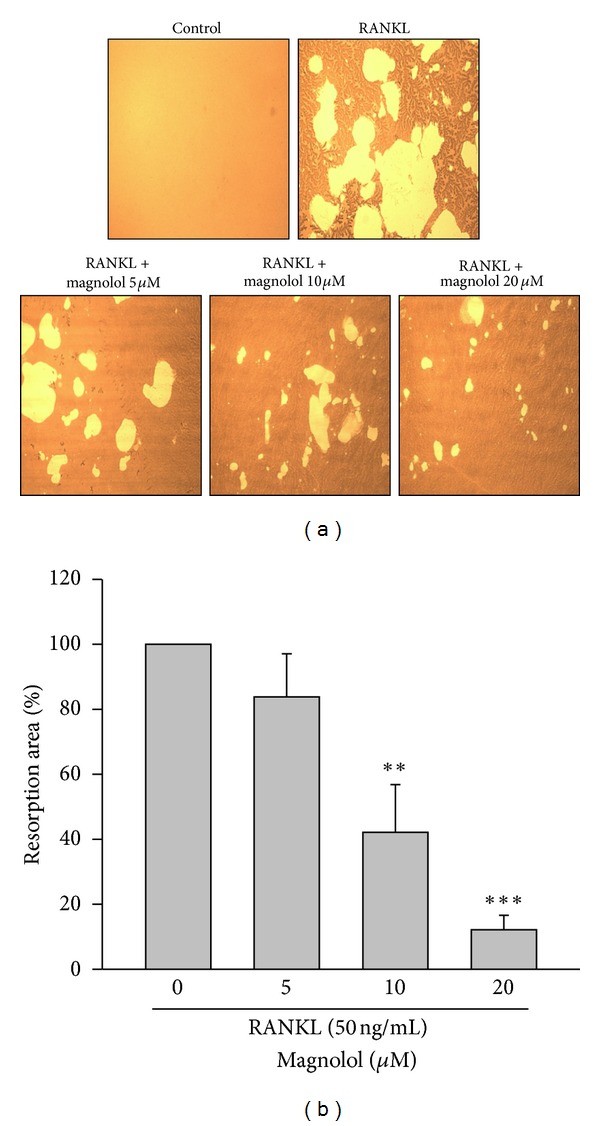
Effect of magnolol on RANKL-induced bone resorption of mature osteoclasts* in vitro. *RAW 264.7 cells were incubated with various concentrations of magnolol (5–20 *μ*M) in the presence of RANKL (50 ng/mL). (a) For the visualization of pits formation, the resorption area was analyzed by a microscope. (b) The percentage of resorption area caused by osteoclasts was measured. Data are expressed as the mean ± SD (*n* = 4). **P < 0.01, ***P < 0.001 versus RANKL-treated alone group.

Next, the effect of magnolol on the resorption of dentin discs by osteoclasts induced by RANKL was examined. A dose-dependent attenuation of the percentage of resorbed areas in the examined slices was observed after treatment with various concentrations of magnolol (Figures 7(a) and 7(b)), suggesting that magnolol could directly abrogate osteoclast-dependent bone resorption in the presence of RANKL.

## 4. Discussion

The oral pathogen-driven inflammation and alveolar bone resorption are important hallmarks of periodontitis. Therefore, targeting to suppress gingival tissue inflammation and periodontal destruction may be a potential strategy to prevent and ameliorate periodontal disease. Currently, the ligature-induced experimental periodontitis is a reliable animal model with site-specific, time-dependent alveolar bone resorption. Our preliminary data has shown that the degree of alveolar bone loss is greatly correlated with the period of ligation confirmed by micro-CT imaging. Thus, too severe with long time (14 days) or too mild with short time (3 days) may be all not suitable for elevation of the effect of drugs, and we found that the 8 days is a better time as previous study [24]. Accordingly, the period of ligation of 8 days was chosen. In the present study, we demonstrated for the first time that administration of magnolol attenuates alveolar bone loss through inhibition of osteoclast formation and the inflammatory responses in ligature-induced periodontitis in rats.

Our results clearly indicated that magnolol exhibits an inhibitory effect on bone resorption confirmed by marked decrease of ACJ-AC distances and improvement of the periodontal histopathological architecture accompanied by reduction of the number of osteoclasts along the bony surface in rats with ligature-induced periodontitis. RANKL is known to play an important role in osteoclastogenesis from osteoclasts precursors to mature osteoclasts, while OPG inhibits osteoclast development and bone resorption [11]. Thus, imbalance between RANKL and OPG contributes to the pathogenesis of periodontitis [28]. To understand the underlying mechanism by which magnolol inhibits osteoclast formation and bone loss, the effect of magnolol on the RANKL/OPG system was investigated. Interestingly, we found that administration of magnolol significantly reduced the RANKL expression without affecting the OPG level, and in turn attenuating RANKL/OPG ratio compared to ligation group. Therefore, inhibition of RANKL expression by magnolol may provide an important mechanism to explain the reduction of osteoclast differentiation, formation, and bone loss in the experimental periodontitis. It is widely recognized that *Porphyromonas gingivalis *and *Aggregatibacter actinomycetemcomitans *are key pathogens in the initiation of periodontal disease, and inhibition of these microorganism accumulation results in a successful outcome in periodontal therapy [3, 29]. Our findings demonstrated that magnolol possesses a direct antimicrobial activity on these periodontopathic microorganisms, which may also involve its protective effect in periodontitis.

A lot of researches have indicated that chronic periodontal disease is associated with a failure of inflammation resolution leading to periodontal tissue destruction [6]. Therefore, modulating the host inflammatory immune responses is an effective way to mitigate periodontal disease progression. During the development of periodontitis, neutrophil recruitment to the site of inflammation is critical to cause periodontal tissue damage by stimulating the local inflammatory mediator production. Moreover, neutrophils are reported to activate osteoclastogenesis in a RANKL-dependent manner [30]. Accordingly, attenuation of MPO activity, a marker of neutrophil infiltration, in the periodontal lesion by magnolol may lead to an inhibition in attenuating subsequent inflammatory responses and bone resorption.

The iNOS-derived NO overproduction is known to play a critical role in the pathogenesis of inflammatory diseases such as periodontitis [31]. It has been reported that higher expression of iNOS is closely related to the pathological characteristics of periodontitis, and blocking iNOS markedly reduces the periodontal tissue injury [32]. Similarly, the inducible COX-2 expression is upregulated in inflamed periodontal tissues [33]. The prostaglandin E_2_ (PGE_2_) derived from arachidonic acid catalyzed by COX-2 has been considered a major inflammatory mediator causing alveolar bone destruction by activating RANKL expression and osteoclastogenesis [34, 35]. On the contrary, blocking COX-2 could effectively inhibit differentiation of osteoclast precursor, RAW264.7 cells, into TRAP-positive (TRAP^+^) osteoclastic cells and the progression of ligature-induced periodontitis in rats [36]. Taken together, these findings strongly support that inhibiting iNOS and COX-2 induction is a promising therapeutic target to attenuate osteoclastogenesis. The present data revealed that the increased iNOS and COX-2 expression in the inflamed gingival tissues of ligated rats were significantly reduced by magnolol. In addition, it has been reported that overproduction of ROS is another crucial factor causing alveolar bone destruction [37]. Consistent with the previous findings [19], we demonstrated that magnolol also exerts a promising antioxidant activity in the experimental periodontitis. Collectively, the protective effect of magnolol in periodontitis may be attributed to downregulation of iNOS and COX-2 as well as ROS formation, and in turn inhibiting alveolar bone loss and inflammatory responses.

It is well known that the MMPs, a family of neutral endopeptidases, are responsible for the degradation of collagen fibers [38] that is the major extracellular matrix component of gingiva. In this study, we found that treatment with magnolol downregulated MMP-1 and MMP-9 but upregulated TIMP-1 in inflamed gingival tissues, suggesting that magnolol has an ability to prevent collagen fiber degradation. As PGE_2_ also acts as an enhancer of collagenase synthesis [39], the inhibition of COX-2-derived PGE_2_ formation by magnolol may involve the reduction of collagen loss. Accordingly, we propose that the favorable effect of magnolol on collagen metabolism may be the result of an attenuation of the degradation of collagen fibers by suppressing endogenous MMPs and/or collagenase production.

Several inflammatory gene expressions including iNOS and COX-2 and production of proinflammatory cytokines and ROS are mediated by NF-*κ*B [26]. Furthermore, NF-*κ*B activation is required for RANKL-induced osteoclast formation and subsequent bone resorption [40]. However, RANKL also activates both classical and alternative pathways of NF-*κ*B in osteoclastic-like cells [40]. To investigate the molecular mechanism involved, the effect of magnolol on NF-*κ*B activation was examined. Under resting state, NF-*κ*B is present in cytoplasm and is bound with an inhibitory molecule I*κ*B-*α*. Upon activation, I*κ*B-*α* is rapidly phosphorylated by I*κ*B kinase complex (IKK) followed by I*κ*B-*α* degradation, which allows NF-*κ*B phosphorylation and translocates into the nucleus to trigger downstream gene induction. In the present study, we demonstrated that magnolol significantly diminished ligature-induced phosphorylation of NF-*κ*B p65 in gingival tissues. The finding suggests that the inhibitory effect of magnolol on inflammatory responses and alveolar bone loss is associated with suppression of NF-*κ*B-mediated processes.

To examine the direct effect of magnolol on osteoclast differentiation, the RAW264.7 murine macrophage cells were cultured with magnolol in the presence of RANKL. Our results showed that magnolol dose dependently decreased the multinucleated (TRAP^+^) osteoclast formation and RANKL-induced TRAP activity of differentiated cells. Similar to the antiresorptive activity of magnolol *in vivo*, magnolol dose dependently inhibited the bone resorptive activity of mature osteoclasts *in vitro* confirmed by a significant reduction of resorption pit area compared to RANKL-treated group alone. In conclusion, the evidence provided by the present study strongly indicates that magnolol can inhibit the inflammatory responses and alveolar bone loss in ligature-induced experimental periodontitis by decreasing periodontopathic microorganism accumulation, NF-*κ*B-mediated inflammatory mediator synthesis, and osteoclastogenesis-related molecules (RANKL) formation. These activities support that magnolol can be considered as a potential therapeutic agent to ameliorate periodontal disease.
